# Biologic switching patterns among children with non-systemic juvenile idiopathic arthritis

**DOI:** 10.1186/s12969-023-00897-6

**Published:** 2023-09-23

**Authors:** Mia Lindegaard Pedersen, Amalie Neve-Græsbøll, Troels Herlin, Mia Glerup

**Affiliations:** Department of Paediatrics and Adolescent Medicine, Aarhus University Hospital, Palle Juul-Jensens Blvd. 99, Aarhus N, 8200 Denmark

**Keywords:** Juvenile idiopathic arthritis, Biologics, Switching, Anti-TNF

## Abstract

**Background:**

In juvenile idiopathic arthritis (JIA) clinical remission is unattainable in some patients despite modern biologic disease-modifying antirheumatic drugs (bDMARD) therapy and switching bDMARD is required. The best choice of second-line bDMARD remains unclear. This retrospective observational study aims to describe the pattern, timing, frequency, and reasons for bDMARD switching among children diagnosed with non-systemic JIA.

**Methods:**

Patients were identified by combining unique personal identification numbers, the International Code of Diagnosis (ICD10) for JIA and biologic therapy. Clinical characteristics were collected retrospectively from the electronic medical records. Included were 200 children diagnosed with non-systemic JIA initiating their first biologic drug between January 1st, 2012, and March 1st, 2021. We compared characteristics of non-switchers vs switchers and early switchers (≤ 6 months) vs late switchers (> 6 months).

**Results:**

The median age at diagnosis was 7.7 years. We found that 37% switched to a different bDMARD after a median age of 6.3 years after diagnosis. In total, and 17.5% of patients switched at least twice, while 6% switched three or more times. The most common reason for switching was inefficacy (57%) followed by injection/infusion reactions (15%) and uveitis (13%). 77% were late switchers, and switched primarily due to inefficacy. All patients started a tumor necrosis factor inhibitor (TNFi) as initial bDMARD (Etanercept (ETN): 49.5%, other TNFis: 50.5%). The patients who started ETN as first-line bDMARD were more likely to be switchers compared to those who started another TNFi.

**Conclusion:**

During a median 6.3-year follow-up biologic switching was observed in more than one third, primarily due to inefficacy.

## Key points

1. Biologic switching was common and found in more than one third of the patients

2. The reason for switching was mainly inefficacy

3. More than 90% switched to a second TNFi

## Background

Juvenile idiopathic arthritis (JIA) is the most common rheumatic disease with the onset in childhood [1, 2]. JIA is an umbrella term of chronic arthropathies [3] which, if left untreated, may lead to severe disability and impaired health-related quality of life. Since JIA is a chronic disease [4] with no available curative drug, the overall goal of treatment is to achieve remission or at least minimal disease activity, to prevent joint damage enabling normal development and growth of the child [2, 5].

The advent of biologic disease-modifying antirheumatic drugs (bDMARDs) targeting specific cytokines or intercellular interactions at the beginning of this millennium has revolutionized the outcome of JIA [6]. A growing number of bDMARDs have now proven their efficacy in a number of randomized controlled trials [7–11]. According to the 2011 American College of Rheumatology (ACR) guidelines, tumor necrosis factor alpha inhibitors (TNFis) are recommended for non-systemic JIA as an add-on treatment when moderate or high disease activity is present three months after initiation of conventional synthetic DMARDs (csDMARDs), or in case of persistent low disease activity six months after initiation of csDMARDs [12, 13]. The recommendations have recently been revised, pointing out that bDMARDs may be appropriate as initial therapy (skipping the prior csDMARD) in children with polyarthritis and involvement of high-risk joints and/or high disease activity [13, 14].

The ultimate treatment target in JIA is remission with sustained disease control. However, not all patients respond to the first bDMARD requiring a switch to other bDMARDs as recommended in the ACR guidelines [12] that were largely based on low or moderate level of evidence. The concept of treat-to-target (T2T) was first implemented for adult rheumatoid arthritis patients defining targets as remission or low disease activity scores [15] and recommendations by a task force have now been reported defining a T2T strategy for JIA [16]. Although still in its early age in pediatric rheumatology the T2T strategy has been increasingly implemented [17–19] but as no head-to-head comparisons between bDMARDs have been attempted, there is no evidence of the superiority of one agent over the other when the target of inactive disease is not reached.

The best choice of a second bDMARD when the initial bDMARD fails remains unclear, and data based on a randomised-controlled trial and real-life experience regarding switching patterns are scarce. Few studies have described patterns of biological switching in JIA patients [20–24], of which only three studies within the past few years [22–24]. In this retrospective observational study, we aimed to describe the pattern, timing, frequency, and reasons for biological switching in Danish patients with non-systemic JIA having free access to an integrated tax-paid healthcare system.

## Patients and methods

We included patients with JIA treated at the pediatric rheumatology department, Aarhus University Hospital (AUH), who received biological treatment from January 1^st^ 2012 until September 1^st^ 2021. We excluded patients who were not biological-naïve at the beginning of our time period, as well as patients who started biological treatment after March 1^st^ 2021, to ensure a minimum of 6 months of follow-up.

From the electronic medical charts, the patients were identified combining the ICD10 codes for JIA (M08.0-M09.0, excluding systemic JIA (M08.2)) and the code for each second-line agent used including the TNFis: etanercept (ETN), adalimumab (ADA), golimumab (GOL), infliximab (IFX), and the non-TNFis: tocilizumab (TCZ), abatacept (ABC), tofacitinib (TCB), and baricitinib. Collection of data included age, gender, JIA category according to ILAR classification, date of diagnosis, uveitis including date of diagnosis, comorbidities (e.g. chronic nonbacterial osteomyelitis (CNO), inflammatory bowel disease (IBD)), date of last clinical visit, medical history regarding csDMARD (methotrexate and leflunomide) and any of previous medical history of bDMARDs, number of active joints before initiation of every biologic therapy, and reasons for switching.

We collected data from each appointment at the clinic, until last follow-up in pediatric rheumatology care or transition to the adult rheumatology clinic when turning 18 years. Patients were considered switchers if they were prescribed more than one bDMARD from January 1^st^ 2012 to September 1^st^ 2021, not including switching of biosimilars. Early switching was considered if switching occurred within 6 months, and late switching was defined if switching occurred after 6 months.

Reasons for switching were recorded as: inefficacy/disease flare, injection/infusion-reaction, severe adverse events, psychological factors, non-compliance, uveitis, IBD, and others.

Inefficacy of treatment was defined by the clinical presentation of one or more active joints or by active uveitis confirmed by an ophthalmologist defined by the SUN criteria [25]. Severe adverse events were defined as severe allergic reactions or thrombocytopenia. An example of psychological factors could be children who refused to receive treatment because of fear of needles.

### Statistical evaluation

Using descriptive statistics we compared characteristics of different groupings in our study population, including switchers and non-switchers. Results were reported as absolute frequencies or expressed as median values with an interquartile range (IQR) in parenthesis. Statistical analysis was performed using IBM SPSS Statistics (version 24). Differences in numerical data between two groups were compared using the Mann-Whitney U test. Group differences in categorical data were analyzed using Pearson’s Chi2-test. All reported *p*-values were based on two-tailed tests for significance, and the level of statistical significance was set at *P* < 0.05.

## Results

**Fig. 1:**
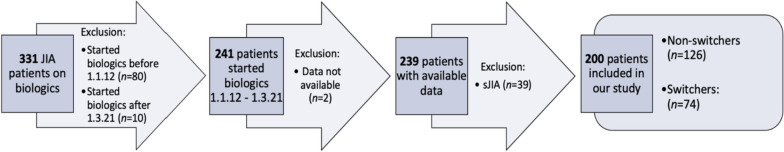
The inclusion of our study cohort

We found 331 patients with JIA treated with biologics during the inclusion period. We excluded 80 patients, who had started bDMARDs before 2012 and accordingly were not bDMARD-naïve at the time of inclusion and excluded 10 patients who had been treated for less than 6 months. Two patients moved to another region during their treatment resulting in unavailable data and were excluded. Lastly, we excluded 39 patients diagnosed with systemic JIA, ending up with a final cohort of 200 children with non-systemic JIA who met the inclusion criteria (Fig. 1).

The characteristics of switchers and non-switchers are listed in Table 1. A total of 1413 person-years of observed follow-up was available, and the median duration of follow-up was 6.3 (3.8–9.4) years (Table 1). Overall, the cohort was predominantly females (71.5%) with extended oligoarticular JIA (30.5%) and RF-negative polyarticular JIA (29.5%). Comparing the group of switchers with non-switchers we found no significant differences among age, gender and JIA subcategories. The switchers had a significantly longer disease duration, but a shorter treatment duration with the first bDMARD (Table 1). At the start of the first bDMARD, both groups had a median of 2 active joints, but the number of cumulative joints was significantly lower among switchers compared to non-switchers (*p* < 0.001).

**Table 1:** Clinical characteristics of patient cohort, comparing switchers and non-switchers

	**All patients (*n* = 200)**	**Switchers (*n* = 74)**	**Non-switchers (*n* = 126)**	*P*-values*
**Females, n (%)**	143 (71.5)	57 (77)	86 (68)	0.185
**Subcategories, n (%)**				
**Persistent oligoarticular**	26 (13)	10 (13.5)	16 (13)	0.549
**Extended oligoarticular**	61 (30.5)	27 (36.5)	34 (27)	
**RFpos-polyarticular**	8 (4)	2 (3)	6 (5)	
**RFneg-polyarticular**	59 (29.5)	23 (31)	36 (29)	
**Psoriatic arthritis**	13 (6.5)	4 (5)	9 (7)	
**Enthesitis related arthritis**	24 (12)	5 (7)	19 (15)	
**Undifferentiated**	9 (4.5)	3 (4)	6 (5)	
**Age at diagnosis,** median years (IQR)	7.7 (2.4–11.8)	6.8 (2.5–10.6)	8.4 (2.4–12.2)	0.146
**Disease duration,** median years (IQR)	6.3 (3.8–9.4)	7.3 (4.7–10.6)	5.4 (3.5–9.0)	0.017
**Disease duration before 1st bDMARD,** median months **(**IQR)	23.1 (5.4–60.4)	30.8 (4.9–61)	18.5 (5.6–60.4)	0.596
**Treatment duration of 1st bDMARD,** median months **(**IQR)	23.9 (10.3–37.3)	13.6 (6.5–32.6)	26.4 (15.4–40.0)	< 0.001
**Active joints at start of 1st bDMARD,** median (IQR)	2 (1–4)	2 (1–4)	2 (1–4)	0.987
**Cumulative active joints,** median (IQR)	6 (2–9)	3 (1–9)	6 (4–10)	< 0.001
**csDMARD before 1st bDMARD, n (%)**	176 (88)	69 (93)	107 (85)	0.042
**csDMARD + 1st bDMARD, n (%)**	155 (77.5)	55 (74)	102 (81)	0.128
**1st bDMARD, n (%)**				
**- Etanercept**	99 (49.5)	49 (66)	50 (40)	< 0.001
**- Adalimumab**	76 (38)	15 (20)	61 (48)	
**- Golimumab**	18 (9)	4 (6)	14 (11)	
**- Infliximab**	7 (3.5)	6 (8)	1 (1)	
**Comorbidities, n (%)**				
**- Uveitis**	44 (22)	20 (27)	24 (19)	0.188
**- CNO**	5 (2.5)	3 (4)	2 (2)	0.281
**- IBD**	3 (1.5)	2 (3)	1 (1)	0.284

*IQR* 1^st^-3^rd^ interquartile range, *bDMARD* Biological disease-modifying antirheumatic drugs, *csDMARD* Conventional synthetic disease-modifying anti-rheumatic drugs, *CNO* Chronic nonbacterial osteomyelitis, *IBD* Inflammatory bowel disease

^*^P-value for comparison of switchers to non-switchers, by Pearson’s Chi2 for categorical variables and Mann-Whitney U-test for continuous variables

**Fig. 2:**
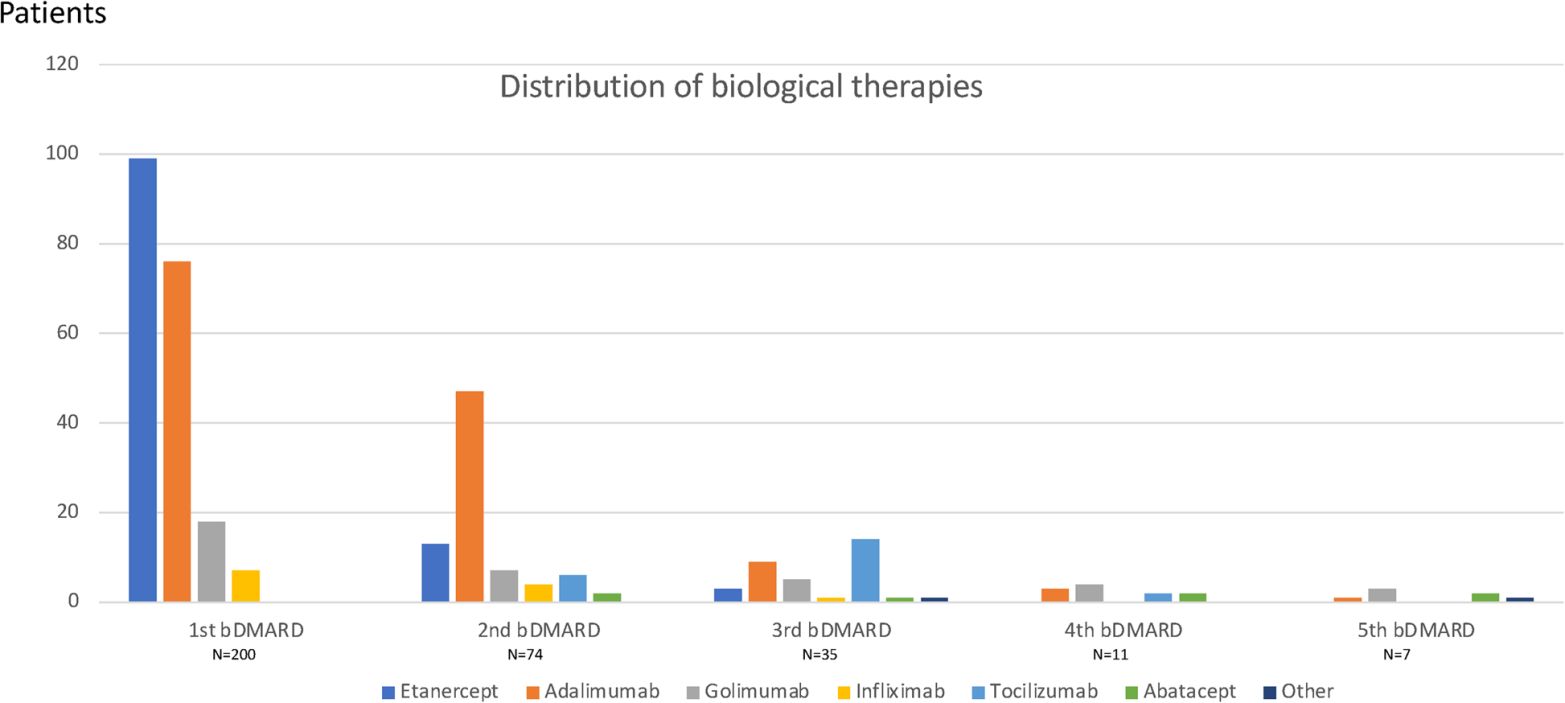
Distribution of the 1^st^ to the 5^th^ biologic disease-modifying antirheumatic drugs (*n* = 200)

The majority (88%) had been treated with methotrexate (176/200) and of these 12% (21/176) had been switched to leflunomide before the start of the initial bDMARD. The percentage of switchers starting directly on biologics without an initial csDMARD was 7%, while the percentage of non-switchers starting directly on biologics was 15% (Table 1). A higher percentage had been treated with csDMARDs in the switcher group compared to the non-switcher group (*p* = 0.042). All 200 patients started with TNFi as the first bDMARD, and most often in combination with csDMARDs (77.5%) (Table 1). During the observation period 74 patients (37%) switched to a second bDMARD, of which 35 (17.5%) switched at least twice, and 12 (6%) switched three times or more (Fig. 2). Of the 74 patients who switched from their first bDMARD, it was shown that 69 (93%) switched to a second TNFi, when the initial TNFi failed, while five patients (7%) switched to a non-TNFi bDMARD (Fig. 2). The five patients who switched to a non-TNFi had a higher incidence of switches (median 3.5 (2.75–4)) compared to those who switched from one TNFi to another (median 2 (1–4)).

Almost half of the patients had started with ETN as the first bDMARD and the rest started with another TNFi (Table 2). The number of switchers was significantly higher in the ETN group compared to the other group (Table 2), where 22% switched at least twice, and 8% at least three times, compared to 13% and 4%, respectively, in the other group. There was no significant difference between early and late switchers in terms of the ETN group compared to the other group (*p* = 0.73). Patients that started with ETN as the first bDMARD were significantly younger at diagnosis (5.0 years compared to 9.7 years, *p* < 0.001) and had significantly longer disease duration than those who had started another TNFi as first line bDMARD (7.3 years vs 4.9 years, *p* = 0.001). Furthermore, the use of csDMARD before introduction of bDMARDs was significantly higher among those on ETN than other TNFis (*p* = 0.003) (Table 2).

**Table 2:** Characteristics of first prescribed bDMARD, comparing etanercept and other TNFis

**Biologic**	**Etanercept**	**Other TNFi**	*P*-value*
**N**	99	101	
**Age at diagnosis,** median years (IQR)	5.0 (2.3–9.9)	9.7 (3.4–13.7)	< 0.001
**Disease duration,** median years (IQR)	7.3 (4.7–10.1)	4.9 (3.3–8.7)	0.001
**Active joints at start of 1st bDMARD,** median (IQR)	2 (1–4)	2 (1–3)	0.592
**Cumulative active joints,** median (IQR)	6 (3–11)	5 (2–7)	0.093
**Switchers*, n (%)***	49 (49)	25 (25)	< 0.001
**0 switch**	50 (51)	76 (75)	0.014
**1 switch**	27 (27)	12 (12)	
**2 switches**	14 (14)	9 (9)	
**≧3 switches**	8 (8)	4 (4)	
**Reasons for 1st switch, n (%)**			
**Inefficacy**	25 (51)	17 (68)	0.164
**Other reasons than inefficacy**^**a**^	24 (49)	8 (32)	
**Disease duration before 1st bDMARD,** median months (IQR)	27.5 (5.6–71.3)	21.5 (5.1–51.9)	0.484
**Treatment duration of 1st bDMARD,** median months (IQR)	24.8 (7.6–44.7)	22.7 (11.7–31.8)	0.540
**- Switchers**	10.2 (5.8–31.7)	16.2 (8.4–33.7)	0.275
**- Non-switchers**	35.1 (21.6–51.9)	24.1 (14.0–30.9)	0.002
**csDMARD before 1st bDMARD, n (%)**	92 (93)	81 (80)	0.003
**csDMARD + 1st bDMARD, n (%)**	79 (80)	79 (78)	0.559

*bDMARD* Biological disease-modifying antirheumatic drugs, *TNFi* Tumor necrosis factor inhibitor, *IQR* 1^st^-3^rd^ interquartile range, *csDMARD* Conventional synthetic disease-modifying anti-rheumatic drugs

^*^P-value for comparison of switchers to non-switchers, by Pearson’s Chi2 for categorical variables and Mann-Whitney U-test for continuous variables

^a^Other reasons for switching than inefficacy included injection/infusion reactions, severe adverse events, psychological factors, non-compliance, uveitis, IBD

The most common reason for the first switch was inefficacy (57%), followed by injection/infusion reactions (15%), uveitis (13%) and other reasons (7%), which included severe psoriatic disease (*n* = 2) and downscaling the method of administration from intravenous to subcutaneous (*n* = 3). Inefficacy became an even more predominant reason to switch when looking at the second (74%), third (67%), fourth (87%), and fifth switch (100%) (Table 3). Among the 49 switchers with ETN as their initial bDMARD, 51% switched due to inefficacy and 49% due to non-inefficacy reasons. The reasons for switching among those who had started with a different TNFi as their initial bDMARD were due to inefficacy in 68%, however, this was not statistically significant (*p* = 0.164) (Table 2). Of those who switched to a non-TNFi as the second bDMARD half of them switched due to inefficacy (data not shown).

**Table 3:** Reasons for switching among all switchers

**Reason for switching**	**1st switch** ***n (%)***	**2nd switch** ***n (%)***	** ≥ 3 switches** ***n (%)***
**Inefficacy**	42 (57)	26 (74)	16 (76)
**Injection/infusion reactions**	11 (15)	3 (9)	3 (14)
**Severe adverse reactions**	2 (3)	1 (3)	2 (10)
**Psychological effects**	2 (3)		
**Non-compliance**	1 (1)	1 (3)	
**Uveitis**	10 (13)	3 (9)	
**IBD**	1 (1)		
**Other reasons**	5 (7)	1 (3)	
**In total**	74 (37)	35 (17.5)	21 (10.5)

In Table 4, the characteristics of patients switching a bDMARD early on (bDMARD treatment for less than 6 months) is compared with patients switching late (bDMARD treatment for more than 6 months). The majority of patients that required switching of bDMARDs were late switchers (77%). Early switchers had significantly shorter disease duration (5.5 years versus 7.9 years, *p* = 0.019). Disease duration before start of first bDMARD was shorter for early than late switchers, however, the difference did not reach significance (*p* = 0.066). We found no significant difference in gender, age at diagnosis, number of active or cumulative joints and the frequency of csDMARD use prior to bDMARD treatment when comparing early and late switchers. However, concomitant use of csDMARD and bDMARD at the time of the switch was more common in early compared to late switchers (83% vs 71%, *p* = 0.019). Active uveitis at the start of initial bDMARD was only present in the late switchers’ group (*n* = 6).

**Table 4:** Characteristics of 1st switch comparing early and late switchers

	**Early switchers (*n* = 17)**	**Late switchers (*n* = 57)**	***P*-values**
**Females (%)**	13 (77)	44 (77)	0.950
**Subcategories, n (%)**			
**Persistent oligoarticular**	3 (18)	7 (12)	0.617
**Extended oligoarticular**	5 (29)	22 (39)	
**RFpos-polyarticular**	1 (6)	1 (2)	
**RFneg-polyarticular**	6 (35)	17 (30)	
**Psoriatic arthritis**	-	4 (7)	
**Enthesitis related arthritis**	2 (12)	3 (5)	
**Undifferentiated**	-	3 (5)	
**Reasons for 1st switch, n (%)**			
**- Inefficacy**	6 (35)	36 (63)	0.101
**- Non-inefficacy reasons**	11 (65)	21 (37)	
**Age at diagnosis,** median years (IQR)	6.1 (3.4–12.7)	6.9 (2.4–10.5)	0.338
**Disease duration,** median years (IQR)	5.5 (3.0–8.7)	7.9 (5.2–10.9)	0.019
**Disease duration before 1st bDMARD,** median months (IQR)	4.1 (2.6–59.1)	33.4 (12.3–61.6)	0.066
**Treatment duration of 1st bDMARD,** median months (IQR)	3.4 (1.9–5.4)	17.8 (8.7–35.1)	< 0.001
**Active joints at start of 1st bDMARD,** median (IQR)	3 (1–7)	2 (1–4)	0.176
**Cumulative active joints,** median (IQR)	2 (1–9)	4 (2–8.5)	0.355
**csDMARD before 1st bDMARD, n (%)**	15 (88)	54 (95)	0.617
**csDMARD + 1st bDMARD, n (%)**	55 (83)	40 (71)	0.019
**1st bDMARD, n (%)**			
**- Etanercept**	13 (76.5)	36 (63.2)	0.733
**- Adalimumab**	2 (11.8)	13 (22.8)	
**- Golimumab**	1 (5.9)	3 (5.3)	
**- Infliximab**	1 (5.9)	5 (8.8)	
**Comorbidities, n (%)**			
**- Uveitis**	4 (24)	16 (28)	
**- CNO**		3 (5)	
**- IBD**		2 (4)	

*IQR* 1^st^-3^rd^ interquartile range, *bDMARD* Biological disease-modifying antirheumatic drugs, *csDMARD* Conventional synthetic disease-modifying anti-rheumatic drugs

^*^P-value for comparison of switchers to non-switchers, by Pearson’s Chi2 for categorical variables and Mann-Whitney U-test for continuous variables

Reasons for switching were differently distributed between the two groups. Two-thirds of late switchers switched due to inefficacy, and one-third due to non-inefficacy reasons (63% vs 37%). The opposite was seen in the group of early switchers, where almost two-thirds switched due to non-inefficacy reasons and one-third due to inefficacy (65% vs 35%), (*p* = 0.101) (Table 4). When looking at the distribution of the first bDMARD between the two groups, the early switchers more often started with ETN (77%) compared to the late switchers (63%). Conversely, the late switchers more often started ADA as the initial bDMARD (23%) than early switchers (12%), but the differences were not statistically significant.

## Discussion

Ever since the advent of the biological therapies remarkable effects on the outcome of moderate to severe JIA have been perceived. Nevertheless, the remaining important question has been whether the treatment efficacy remains to achieve sustained remission. In this retrospective study using real-world data we found that 37% of patients with non-systemic JIA switched from their initial bDMARD to a different bDMARD with median 75 months of follow-up. We saw a higher switching frequency than was recently reported from British and American data registry studies [22, 23]. They found that 23% and 26%, respectively, switched to a second biologic. We also found that 17.5% of patients switched at least twice and 6% of the patients switched three or more times, which again are higher percentages than observed in the study from UK (5% and 1%, respectively, [22]). Likely, this difference is due to our median follow-up time being 75 months, which is remarkably longer in comparison with 26 and 30 months in the two studies [22, 23]. Notably, the ACRPed70 response to TNFi in previous RCT studies of polyarticular JIA reached no more than 36–66% during the first 3–9 months of treatment [7–10]. In addition, similar studies on rheumatoid arthritis suggest that 30–40% switch within the first year of treatment initiation which increases up to 50% at 2 years [27, 28]. Considering this, we do not regard 37% as an unusual high switching frequency of the initial bDMARD.

We chose only to include patients who started biologic treatment after 2012. Previous relevant studies [22–24] were based on data collected starting from 2007–2010. Since then, the panel of bDMARDs approved for JIA has expanded, which might contribute to the lower percentages of switches in the three previous studies. Furthermore, the absence of an evidence-based threshold for lack of efficacy guiding physicians may be the occasion of international differences and subjectivity in the proclivity to switch patients. Other practice-associated factors may also be associated with this effect, e.g. insurance status which is not a dispute in a Danish, publicly financed health care system.

The data on which we have based our study is extracted from the electronic patient file containing detailed logs on all visits and prescribed medications, giving us detailed information about reasons for switching, disease activity, dates of starting or ending treatment. This is different from the previously reported British and American studies based on data registries. When comparing the group of switchers with non-switchers we found that the switchers had a significantly longer disease duration, and a higher occurrence of csDMARD treatment prior to bDMARD. However, we found no significant differences among age, gender, and JIA subcategories. Unexpectedly, we showed that non-switchers had more cumulative affected joints than switchers, 6 vs 3 affected joints, *p* < 0.001.

All patients started a TNFi as the initial bDMARD, and 93% of switchers were prescribed an intra-class switch as the second bDMARD. When distinguishing between ETN or another TNFi as the initial bDMARD, the percentage of switchers was higher among the ETN group. This suggests that having another TNFi than ETN as the initial bDMARD diminishes the likelihood of switching bDMARDs. In addition to this, we found it statistically significant that patients on ETN as the initial bDMARD were younger at time of diagnosis, more often had used csDMARD before starting bDMARD and had a longer disease duration than those starting other TNFis. It is unknown whether this difference causes the higher number of switches because of a longer follow-up, or reversely, whether the longer disease duration could be due to those on ETN having a higher frequency of switches, extending their disease course.

Inefficacy was the main reason for switching from the initial bDMARD to a second and was observed in 57%. This was in congruence with the previous studies by Kearsley-Fleet et al. and Mannion et al., showing inefficacy in 60% and 58%, respectively [22, 23]. When differentiating between etanercept and other TNFis, we found that 51% of the switchers on ETN switched due to inefficacy and 49% due to other reasons like intolerance, while 68% of the switchers on other TNFis switched because of inefficacy and 32% due to other reasons. The higher percentage of switching because of other reasons than inefficacy in the ETN group could be explained by a younger age at diagnosis and by having difficulties accepting the injections twice a week.

Patients on initial bDMARD for no longer than 6 months were designed as early switchers (23%) and 77% were late switchers on first bDMARD (> 6 months). Early switchers switched more often due to non-inefficacy reasons than inefficacy (65% vs 35%) in comparison to late switchers who conversely switched more often due to inefficacy (63% vs 37%), however, the difference was not statistically significant (*p* = 0.101). Opposite our results Mannion et al. found that 75% switched within 6 months of treatment and 25% after 6 months. Also, regarding reasons for switching their results directly differ from ours with 67% of early switchers switching due to inefficacy and only 31% of late switchers switching due to inefficacy.

Another characteristic associated with an early switcher, although it did not reach statistical significance (*p* = 0.06), was a long disease duration before the start on biologic treatment. If this tendency could be confirmed in another larger study, this could advocate for starting biologic treatment sooner rather than late in a disease course to avoid an early switch in treatment and possibly gain a better outcome.

It may be seen as a limitation that our study has a retrospective, single-center design. All decisions in this single center study regarding the individual biological therapy has been made according to the guidelines approved by the Danish national health authorities which again has been largely based on the ACR 2011 recommendations [12]. Thus, the decisions made by the small group of pediatric rheumatologists collaborating closely together evaluating and discussing patients’ disease courses and treatments, have resulted in relatively homogeneous interpretations and treatment decisions. However, the retrospective design of the study leaves uncertainty regarding whether all patients strictly adhere to the definitions of remission, flare and treatment ineffectiveness. Another limitation is that our results may not be generalizable to populations outside Denmark, e.g. countries where insurance restrictions strongly affect the use of biologics.

Our study has some strengths to be mentioned. We used data collected through 2012 and onwards to ensure a treatment strategy and a bDMARD availability comparable as of today. The data used are extracted from the electronic medical records with mandatory recruitment minimizing missing data.

## Conclusion

Biologic switch has an increasing trend due to new biologic agents on the market and an expanding use of the treat-to-target strategy. We found that 37% of patients with non-systemic JIA switched bDMARD once and 17.5% switched at least twice within 75 months of follow-up. Lack of efficacy was the most common reason for switching. As no head-to-head comparisons between these second biologics have been achieved, there is no evidence on the superiority of one biologic agent over the other when switching. Additionally, identification and validation of biomarkers that will present an ability to predict response to different biologic treatments is yet an unmet need.

## Data Availability

The datasets generated and/or analysed during the current study are available from the correspondig author on reasonable request.

## Abbreviations

ABC: Abatacept
ACR: American College of Rheumatology
ADA: Adalimumab
AUH: Aarhus University Hospital
bDMARD: Biologic disease-modifying anti-rheumatic drug
CNO: Chronic nonbacterial osteomyelitis
csDMARD: Conventional synthetic disease-modifying anti-rheumatic drug
ERA: Enthesitis-related arthritis
ETN: Etanercept
GOL: Golimumab
IBD: Inflammatory bowel disease
ICD10: International Code of Diagnosis
IFX: Infliximab
IQR: Interquartile range
JIA: Juvenile idiopathic arthritis
T2T: Treat-2-target
TCB: Tofacitinib
TCZ: Tocilizumab
TNFi: Tumour necrosis factor inhibitor
